# Correlates of alcohol consumption among Germans in the second half of life. Results of a population-based observational study

**DOI:** 10.1186/s12877-017-0592-3

**Published:** 2017-09-08

**Authors:** André Hajek, Jens-Oliver Bock, Siegfried Weyerer, Hans-Helmut König

**Affiliations:** 1 Department of Health Economics and Health Services Research, Hamburg Center for Health Economics, University Medical Center Hamburg-Eppendorf, Hamburg, Germany; 2 Central Institute of Mental Health, Medical Faculty Mannheim/Heidelberg University, Mannheim, Germany

**Keywords:** Alcohol, Psychological factors, German Ageing Survey

## Abstract

**Background:**

Several studies have investigated the predictors of alcohol consumption behavior among adolescents and young adults. However, the body of evidence about the relationship between in particular psychological factors and alcohol consumption among individuals in the second half of life is still limited. Hence, we aimed at identifying factors associated with alcohol consumption among individuals aged 40 and above, especially focusing on psychological correlates.

**Methods:**

Data were derived from a population-based sample of community-dwelling individuals aged 40 to 95 years (*n* = 7820) in Germany. Alcohol consumption was rated as ‘never’ (never drinkers), ‘rarer than once a month’, ‘one to three times a month’, ‘once a week’, ‘several times a week’ (occasional drinkers), and ‘daily’ (daily drinkers). Socio-economic factors, the illness level and physical activity were considered as possible determinants of alcohol consumption. In addition, positive and negative affect, life satisfaction, optimism, self-esteem, self-efficacy, and self-regulation were included as psychological factors. Multinomial regressions were used to identify factors associated with drinking behavior.

**Results:**

12.0% of the individuals were daily drinkers, 76.5% were occasional drinkers, and 11.5% of the individuals never drank alcohol. After adjusting for various potential confounders, multinomial logistic regressions revealed that, compared with never drinking, occasional and daily drinking were positively associated with a decreased loneliness, a higher life satisfaction, a higher positive affect, a higher optimism, a higher self-efficacy (occasional drinkers), a higher self-esteem, and less perceived stress. In addition, occasional and daily drinking were positively associated with less physical illnesses, male gender, and income as compared with never drinking.

**Conclusions:**

The current study extends the existing literature on alcohol consumption behavior by new insights of correlates of drinking behavior among individuals in the second half of life. Since interventions are available to address this risk factor, this might help to identify individuals with increased alcohol consumption.

**Electronic supplementary material:**

The online version of this article (10.1186/s12877-017-0592-3) contains supplementary material, which is available to authorized users.

## Background

Alcohol consumption is associated with a tremendous burden of chronic disease and disability [1]. For example, worldwide 3.3 million deaths every year are caused by harmful use of alcohol, which equally leads to various non-communicable diseases, injuries, and infectious diseases such as tuberculosis [2].

Risk factors of alcohol consumption and risky alcohol use have been extensively studied. For example, it has been shown that undesirable life events, e.g., divorce, are associated with increased alcohol consumption [3]. Furthermore, a substantial body of evidence has found that psychological factors are associated with alcohol consumption among *adolescents and young adults* [4–9]. For example, it has been shown that self-regulation was negatively associated with alcohol consumption in adolescents and students [7, 10]. It has also been reported that satisfaction with life is negatively associated with regular alcohol use in adolescents [9].

However, the body of evidence about the relationship between in particular *psychological factors* and *alcohol consumption* among *individuals in the second half of life* is still limited [11–13]. This is despite the fact that several stressful life events occur in the second half of life such as bereavement, social isolation or disability in one’s self or family members, which might be associated with alcohol intake. In addition, because of the demographic ageing in Germany, the number of older people is projected to rise substantially, underlining the meaning of alcohol consumption in old age. Therefore, it is important to study individuals in the second half of life (40 years and over) [14]. Furthermore, in older age, increased or daily alcohol consumption has been found to be associated with numerous adverse health outcomes including insomnia, frequent falls or depression [15]. This is in accordance with, for example, the guidelines of New Zealand [16], suggesting at least two alcohol-free days per week.

Thus, the aim of the current study was to identify factors related to alcohol consumption, particularly focusing on psychological factors, using a population-based sample of individuals aged 40 and over. This lack of knowledge is important to address because it might help to characterize individuals with risky alcohol consumption.

It has been shown that life satisfaction is negatively associated with regular alcohol use in a younger sample [9]. Thus, we hypothesize that life satisfaction as well as positive affect and optimism are negatively associated with alcohol consumption. In contrast, we hypothesize that negative affect and loneliness are positively associated with alcohol consumption. This appears plausible because depressive symptoms (which are strongly associated with negative affect, loneliness and stress) are associated with increased alcohol consumption [17]. A previous study has shown that self-regulation was negatively associated with alcohol consumption [7]. In line with this study, we hypothesize that self-regulation is negatively associated with alcohol consumption. This appears plausible because individuals scoring high in self-regulation delay short-term goals (e.g., drink in a pub) to achieve long-term goals (e.g., stay healthy). Moreover, in accordance with previous findings [18], we hypothesize that self-esteem is negatively associated with alcohol consumption. In addition, it has been demonstrated that increased alcohol consumption is associated with low self-efficacy [19]. In line with these findings, we hypothesize that self-efficacy is negatively associated with alcohol consumption.

## Methods

### Study population

Participants were from the fifth wave (2014) of the nationwide German Aging Survey (DEAS), a large population-based observational study with community-dwelling participants at the age of 40 years and above. The recruitment for the baseline wave took place in 1996; data for further waves were collected in 2002 (second wave), 2008 (third wave), 2011 (fourth wave), and in 2014. The interval between the waves was reduced from 6 to 3 years in order to reduce attrition and to capture midterm changes adequately [20].

National probability sampling was used to determine potential participants via registry offices in order to obtain representative samples for the older German population. The individuals were offered participation via mail.

Each of the follow-up waves comprised longitudinal panel samples with participants who had already taken part before in the study and cross-sectional samples with participants taking part for the first time (except for wave 4, which is a pure panel survey). The DEAS has a cohort-sequential design, linking cross-sectional samples with longitudinal samples.

Nearly 5200 individuals were interviewed in wave 2, 8200 individuals took part in wave 3, approximately 4850 individuals were interviewed in wave 4 and 10,324 individuals took part in wave 5. More than 1526 individuals from baseline were re-interviewed in wave 2. While nearly 2000 individuals were re-interviewed in wave 3, about 6200 individuals took part for the first time. While about 6000 individuals took part for the time in wave 5, more than 4000 individuals were re-interviewed. Further details (e.g., sampling composition) were provided in detail elsewhere [20].

In 2014, the response rates for the panel sample was 61%, for the cross-sectional sample 25%, respectively. Baseline participants who provided written informed consent were contacted again in order to participate in the other waves. Compared with other European surveys, DEAS response rates are rather low [21]. However, the response rates of the DEAS are similar to comparable German surveys [22]. A decrease in participation rates in surveys in Western countries can be observed in the last years. However, this phenomenon is most pronounced in Germany [21]. The DEAS made efforts to alleviate this trend. For example, the incentives for respondents were increased (since 2008: €10). Notwithstanding, response rates for baseline samples decreased from about 50% (1996) to the figure reported above.

For this year, a total of *n* = 7820 individuals took part in the study and provided information on alcohol consumption (gross sample: *n* = 23,984). Alcohol consumption was not assessed in former waves. The survey consisted of a face-to-face computer-assisted personal interview at the participants’ homes and a drop-off questionnaire (*n* = 7952). Written informed consent was obtained from all participants.

### Dependent variable

Alcohol intake (exact wording: How often do have a drink containing alcohol (e.g. beer, wine, sparkling wine, spirits, long drinks) was self-reported as ‘never’ (non-drinkers); ‘rarer than once a month’, ‘one to three times a month’, ‘once a week’, or ‘several times a week’ (collapsed into the category occasional drinkers); and ‘daily’ (daily drinkers). The threshold for a “risky” alcohol intake was considered as daily alcohol consumption (“non-risky”: otherwise) [15]. A time frame (e.g., last 6 months or last year) was not specified. Thus, ex-drinkers would fall into the category of non-drinkers. This will be addressed in the discussion section.

### Independent variables: Psychological factors

Loneliness was measured using a short version of the widely used 11-item De Jong Gierveld Loneliness Scale [23]. It has been proven to be valid [23, 24]. Cronbach’s alpha was .83 in our sample. Further details concerning all psychological measures used in the current study are depicted in Table 1. The well-established Satisfaction with Life Scale (SWLS) [25] was used to assess the satisfaction with life, with Cronbach’s alpha = .86. Positive and negative affect were assessed using the Positive and Negative Affect Schedule (PANAS) [26], which has very good psychometric properties [27]. Cronbach’s Alpha was .87. Optimism was quantified using a validated scale by Brandtstädter and Wentura [28]. Cronbach’s alpha was .84. Using the widely-used scale by Schwarzer and Jerusalem [29], self-efficacy was assessed. Cronbach’s alpha was .75. The well-established Rosenberg scale [30] was used to measure self-esteem. Cronbach’s alpha was .84. Stress was measured using a scale by Cohen et al. [31], with Cronbach’s alpha = .70. According to a scale by Ziegelmann and Lippke [32] which was based on a scale developed by Freund and Baltes [33], self-regulation was operationalized. Cronbach’s alpha was .78.

**Table 1:** Psychological factors. Items and explanations

Psychological factors	Items and explanations
Loneliness (De Jong Gierveld & Van Tilburg, 2006) [23]	Emotional loneliness 1. I experience a general sense of emptiness. 2. I miss having people around. 3. I often feel rejected. Social loneliness 1. There are plenty of people I can rely on when I have problems. (*) 2. There are many people I can trust completely. (*) 3. There are enough people I feel close to. (*) Scale represents the mean of at least 3 required valid items, 3 items have been recoded. 1 = strongly agree 2 = agree 3 = disagree 4 = strongly disagree High values represent high loneliness.
Life satisfaction (SWLS, Pavot & Diener, 1993) [25]	1. In most ways my life is close to my ideal. 2. The conditions of my life are excellent. 3. I am satisfied with my life. 4. So far I have gotten the important things I want in life. 5. If I could live my life over, I would change almost nothing. Scale represents the mean of at least 3 required valid items, all items have been recoded. 1 = strongly agree 2 = agree 3 = neither agree nor disagree 4 = disagree 5 = strongly disagree High values represent high life satisfaction.
Negative affect (PANAS, Watson et al., 1988) [26]	In the following you will find a number of words that describe different feelings and emotions. Please indicate to what extent you have felt this way during the past few months. 1. Distressed 2. Upset 3. Guilty 4. Scared 5. Hostile 6. Irritable 7. Ashamed 8. Nervous 9. Jittery 10. Afraid Scale represents the mean of at least 3 required valid items. 1 = very slightly or not at all 2 = a little 3 = moderately 4 = quite a bit 5 = extremely High values on the NA scale represent high frequency of negative emotions.
Positive affect (PANAS, Watson et al., 1988) [26]	In the following you will find a number of words that describe different feelings and emotions. Please indicate to what extent you have felt this way during the past few months. 1. Enthusiastic 2. Excited 3. Strong 4. Interested 5. Proud 6. Alert 7. Inspired 8. Determined 9. Attentive 10. Active Scale represents the mean of at least 3 required valid items. 1 = very slightly or not at all 2 = a little 3 = moderately 4 = quite a bit 5 = extremely High values on the PA scale represent high frequency of positive emotions.
Optimism (Brandtstädter & Wentura, 1994) [28]	1. I am looking forward to the life ahead of me. (*) 2. For me the future is full of hope. (*) 3. Thinking about my future makes me worry. 4. I look to the future with confidence. (*) 5. The future holds a lot of good in store for me. (*) Scale represents the mean of at least 3 required valid items, 4 items have been recoded. 1 = strongly agree 2 = agree 3 = disagree 4 = strongly disagree High values represent high optimism.
Self-efficacy (Schwarzer & Jerusalem, 1999) [29]	1. It is easy for me to stick to my aims and accomplish my goals. 2. I can usually handle whatever comes my way. 3. I can solve most problems if I invest the necessary effort. 4. If I am in trouble, I can usually think of a solution. 5. When I am confronted with a problem, I can usually find several solutions Scale represents the mean of at least 3 required valid items, all items have been recoded. 1 = strongly agree 2 = agree 3 = disagree 4 = strongly disagree High values represent high self-efficacy.
Self-esteem (Rosenberg, 1965) [30]	1. On the whole, I am satisfied with myself. (*) 2. I am able to do things as well as most other people. (*) 3. All in all, I am inclined to feel that I am a failure. 4. I feel that I have a number of good qualities. (*) 5. I certainly feel useless at times. 6. I feel that I‘m a person of worth, at least on an equal plane with others. (*) 7. I feel I do not have much to be proud of. 8. I take a positive attitude toward myself. (*) 9. I wish I could have more respect for myself. 10. At times I think I am no good at all. Scale represents the mean of at least 3 required valid items, 5 items have been recoded. 1 = strongly agree 2 = agree 3 = disagree 4 = strongly disagree High values represent high self-esteem.
Stress (Cohen et al., 1983) [31]	1. In the last month, how often have you felt that you were unable to control the important things in your life? 2. In the last month, how often have you felt confident about your ability to handle your personal problems? (*) 3. In the last month, how often have you felt that things were going your way? (*) 4. In the last month, how often have you felt difficulties were piling up so high that you could not overcome them? Scale represents the mean of at least 2 required valid items, 2 items have been recoded. 1 = never 2 = seldom 3 = sometimes 4 = often 5 = very often High values represent high perceived stress.
Self-regulation (Freund & Baltes, 2002; Ziegelmann & Lippke, 2006) [32, 33]	1. I do everything I can to realize my plans. (*) 2. I have set my goals clearly and stick to them. (*) 3. When it becomes harder for me to get the same results, I keep trying harder until I can do it as well as before. (*) 4. When I can’t do something important the way I did before, I look for a new goal. (*) Scale represents the mean of at least 2 required valid items, all items have been recoded. 1 = strongly agree 2 = agree 3 = disagree 4 = strongly disagree High values represent high self-regulation.

Items with asterisk have been recoded

The instruments used to quantify the psychological factors in the present study are widely used and accepted. They have also been validated in German language. For example, the scale to quantify optimism has been validated by Brandtstädter and Wentura [28]. Moreover, it has for example been shown that the self-efficacy scale is valid [34].

### Independent variables: Other variables

Moreover, independent variables were included as follows: age, gender, and marital status (married and living together with spouse; others (married and living separately, divorced, widowed, and single), region (West and East Germany) and individual monthly net equivalence income (OECD scale). In addition, physical activity (‘never’, ‘rarer than once a month’, ‘one to three times a month’, ‘once a week’, ‘several times a week’, and ‘daily’) was included. Furthermore, the sum of chronic conditions such as cancer or bad circulation (no; yes) were included, ranging from 0 to 11.

It is widely acknowledged that alcohol intake is strongly associated with depression. However, the causal pathway is not entirely clear and there is evidence that increased alcohol consumption can cause depression [35, 36]. For this reason, depression was solely added to the main model in additional analysis. Depression (sum score ≥ 18) was quantified using the Center for Epidemiological Studies Depression Scale (15 items, 0–45).

Furthermore, in additional analysis, our main regression model was stratified by age (< 65 years; ≥ 65 years) because these two age groups might differ in many ways (e.g. social ties, less structured days in retirement or less work-related activities) [37].

### Statistical analysis

Analyses of variance were used for the continuous variables, and chi-squared tests for all other variables. Multinomial logistic regressions were performed, with (1) non-drinkers (reference category), (2) occasional drinkers, and (3) daily-drinkers. Furthermore, occasional drinking was used as reference category (results not shown, but available upon request). The criterion for statistical significance was set at *p* < .05. All analyses were performed using Stata 14.0 (StataCorp, College Station, Texas, USA). The psychological factors were entered separately in the regression models because they are strongly correlated with each other.

## Results

### Sample characteristics

Table 2 shows the sample characteristics, stratified by drinking status. A total of *n* = 7820 participants provided data on alcohol consumption. 12.0% of these individuals were daily drinkers, 76.5% were occasional drinkers, and 11.5% never drank alcohol. These groups differ significantly from each other regarding all socio-economic variables, the illness level, status of activity, and all psychological factors except self-regulation.

**Table 2:** Characteristics, stratified by alcohol consumption (*n* = 7820)

	Persons who never drink alcohol (*n* = 896; 11.5%)		Persons who drink alcohol at least ‘rarer than once a month’ to ‘several times a week’ (*n* = 5986; 76.5%)		Persons who drink alcohol daily (*n* = 938; 12.0%)		*p*-value †
	N/Mean	%/(SD)	N/Mean	%/(SD)	N/Mean	%/(SD)	
Gender: Female	547	61.1%	3200	53.5%	235	25.1%	*p* < .001
Age in years	66.79	(11.31)	63.77	(11.24)	66.89	(10.47)	*p* < .001
Marital status							*p* < .001
Married and living together with spouse	538	60.2%	4223	70.7%	704	75.1%	
Married, living separated from spouse	13	1.4%	97	1.6%	16	1.7%	
Divorced	117	13.1%	601	10.1%	66	7.0%	
Widowed	159	17.8%	624	10.4%	92	9.8%	
Single	67	7.5%	427	7.2%	60	6.4%	
Monthly net equivalent income (€)	1547.99	(1361.48)	1960.99	(1341.80)	2205.92	(1525.89)	*p* < .001
Region: West Germany	595	66.4%	3988	66.6%	672	71.6%	*p* < .01
Physical activity							*p* < .001
- daily	79	8.8%	501	8.4%	74	7.9%	
- several times a week	164	18.3%	1738	29.0%	232	24.7%	
- once a week	120	13.4%	1136	19.0%	174	18.5%	
- one to three times a month	39	4.4%	468	7.8%	77	8.2%	
- less frequently	89	9.9%	711	11.9%	115	12.3%	
- never	405	45.2%	1431	23.9%	266	28.4%	
Number of physical illnesses	3.19	(2.13)	2.49	(1.84)	2.72	(1.77)	*p* < .001
Loneliness	1.88	(0.60)	1.76	(0.53)	1.78	(0.54)	*p* < .001
Life satisfaction	3.58	(0.84)	3.83	(0.70)	3.84	(0.73)	*p* < .001
Positive affect	3.40	(0.61)	3.57	(0.52)	3.56	(0.52)	*p* < .001
Negative affect	2.13	(0.58)	2.09	(0.52)	2.06	(0.52)	*p* < .05
Optimism	2.80	(0.64)	3.00	(0.53)	2.96	(0.56)	*p* < .001
Self-efficacy	3.00	(0.52)	3.08	(0.42)	3.08	(0.44)	*p* < .001
Self-esteem	3.31	(0.46)	3.41	(0.40)	3.40	(0.40)	*p* < .001
Self-regulation	3.99	(0.59)	3.98	(0.50)	3.96	(0.53)	*p* = .50
Perceived stress	2.55	(0.71)	2.35	(0.64)	2.28	(0.67)	*p* < .001
Depression	122	13.9%	324	5.5%	41	4.5%	*p* < .001

† Analyses of variance were used for the continuous variables, and chi-squared tests for all other variables; Loneliness [23]; Life satisfaction (SWLS, [25]); Positive and negative affect (PANAS, [26]); Optimism [28]; Self-efficacy [29]; Self-esteem [30]; Self-regulation [33]; Perceived stress [31], Depression (CES-D ≥ 18)

The mean age of all participants was 65.5 years; it was higher in the non-drinking and daily drinking group than in the occasional drinking group (post hoc contrasts were performed, but not shown here). About 75% of daily drinkers were male, although the proportion of men was only 53% in the whole sample. Among those who never drank, the proportion of men was only 39%. The status of being married and living together with the spouse increased from non-drinking, to occasional drinking and to daily drinking status. The same applied to income that increased from €1548 (non-drinking) to €2206 (daily drinking) per month. Both non-drinkers and daily drinkers suffered from more chronic physical conditions than occasional drinkers did.

With respect to the psychological factors, loneliness was more pronounced in the daily and non-drinking groups than in the group of the occasional drinkers. Life satisfaction increased with more regular alcohol consumption, negative affect decreased consistently, while positive affect, optimism, and self-esteem was highest in the group of occasional drinkers. The level of perceived stress decreased with increased alcohol consumption. Self-efficacy was higher in the group of occasional drinkers compared with the non-drinking group.

### Regression analysis

Table 3 and Table 4 show results of fully adjusted multinomial logit regression analyses with the drinking status (non-drinking; occasional drinking; daily drinking) as dependent variable, with non-drinking as reference category. For reasons of clarity, the results of multinomial regression analyses were displayed in two tables (Table 3: non-drinking vs. occasional drinking; Table 4: non-drinking vs. daily drinking). Relative risk ratios were reported. It was adjusted for age, gender, marital status, income, region, physical activity and the number of chronic conditions. Based on theoretical considerations and empirical studies, these variables were selected. Thus, stepwise regression models were not used. The psychological factors were entered separately in the regression models. Consequently, nine multinomial regression models were computed.

**Table 3:** Correlates of alcohol consumption. Results of multinomial regressions Part 1 (Occasional drinkers; Reference category: non-drinker; relative risk ratios were reported, 95% CIs in parentheses)

Independent variables	Occasional drinkers	Occasional drinkers	Occasional drinkers	Occasional drinkers	Occasional drinkers	Occasional drinkers	Occasional drinkers	Occasional drinkers	Occasional drinkers
Female (Ref. Male)	0.715***	0.721***	0.725***	0.740***	0.745***	0.739***	0.734***	0.751***	0.727***
	(0.609–0.839)	(0.615–0.846)	(0.618–0.849)	(0.631–0.869)	(0.636–0.872)	(0.631–0.866)	(0.627–0.860)	(0.641–0.881)	(0.620–0.853)
Age	0.992*	0.989**	0.993+	0.993+	0.993+	0.993+	0.992*	0.992*	0.994
	(0.985–1.000)	(0.981–0.996)	(0.986–1.001)	(0.985–1.001)	(0.986–1.001)	(0.985–1.001)	(0.985–1.000)	(0.985–1.000)	(0.987–1.002)
Married, living separated from spouse (Ref.: married, living together with spouse)	1.033	1.106	0.995	0.994	1.025	0.997	0.991	1.027	0.929
	(0.552–1.934)	(0.590–2.071)	(0.533–1.859)	(0.533–1.855)	(0.548–1.916)	(0.534–1.862)	(0.531–1.850)	(0.550–1.916)	(0.497–1.735)
Divorced	0.797+	0.876	0.779*	0.779*	0.806+	0.783*	0.788*	0.781*	0.761*
	(0.629–1.010)	(0.689–1.114)	(0.615–0.986)	(0.616–0.986)	(0.637–1.020)	(0.620–0.990)	(0.623–0.997)	(0.617–0.988)	(0.601–0.962)
Widowed	0.774*	0.777*	0.765*	0.766*	0.766*	0.764*	0.775*	0.774*	0.798+
	(0.616–0.972)	(0.620–0.975)	(0.610–0.960)	(0.611–0.961)	(0.611–0.960)	(0.609–0.957)	(0.619–0.970)	(0.616–0.972)	(0.633–1.005)
Single	0.896	0.977	0.879	0.863	0.884	0.871	0.881	0.881	0.837
	(0.665–1.207)	(0.722–1.320)	(0.653–1.184)	(0.641–1.161)	(0.658–1.188)	(0.648–1.169)	(0.656–1.183)	(0.655–1.186)	(0.623–1.124)
Monthly net equivalent income (in €1000)	1.369***	1.349***	1.383***	1.422***	1.381***	1.401***	1.386***	1.352***	1.401***
	(1.238–1.514)	(1.218–1.494)	(1.248–1.531)	(1.283–1.575)	(1.248–1.528)	(1.266–1.550)	(1.252–1.533)	(1.222–1.495)	(1.266–1.551)
East Germany (Ref. West Germany)	1.205*	1.203*	1.213*	1.226*	1.230*	1.230*	1.215*	1.214*	1.254**
	(1.022–1.420)	(1.022–1.416)	(1.031–1.428)	(1.041–1.444)	(1.046–1.446)	(1.046–1.446)	(1.034–1.429)	(1.031–1.430)	(1.064–1.478)
Physical activity: Several times a week (Ref.: daily)	1.490*	1.476*	1.502**	1.492*	1.482*	1.483*	1.487*	1.489*	1.459*
	(1.096–2.026)	(1.087–2.005)	(1.106–2.039)	(1.100–2.025)	(1.092–2.010)	(1.094–2.012)	(1.097–2.017)	(1.095–2.025)	(1.070–1.989)
Once a week	1.428*	1.435*	1.500*	1.445*	1.434*	1.436*	1.432*	1.450*	1.381+
	(1.035–1.971)	(1.041–1.978)	(1.088–2.067)	(1.049–1.990)	(1.042–1.974)	(1.043–1.975)	(1.040–1.970)	(1.051–2.002)	(0.999–1.909)
One to three times a month	1.686*	1.650*	1.714*	1.679*	1.694*	1.674*	1.692*	1.719*	1.656*
	(1.103–2.577)	(1.084–2.514)	(1.126–2.610)	(1.103–2.555)	(1.113–2.578)	(1.100–2.547)	(1.112–2.574)	(1.125–2.627)	(1.081–2.536)
Less frequently	1.144	1.157	1.196	1.148	1.173	1.157	1.165	1.157	1.141
	(0.810–1.615)	(0.820–1.632)	(0.848–1.688)	(0.814–1.619)	(0.832–1.654)	(0.821–1.631)	(0.826–1.641)	(0.819–1.633)	(0.805–1.617)
Never	0.667**	0.689**	0.725*	0.674**	0.704*	0.675**	0.682**	0.692*	0.652**
	(0.504–0.883)	(0.520–0.911)	(0.547–0.961)	(0.510–0.891)	(0.532–0.931)	(0.511–0.892)	(0.516–0.902)	(0.523–0.918)	(0.491–0.867)
Number of physical illnesses	0.901***	0.917***	0.907***	0.894***	0.914***	0.899***	0.905***	0.909***	0.889***
	(0.865–0.939)	(0.880–0.956)	(0.871–0.945)	(0.857–0.932)	(0.877–0.953)	(0.863–0.936)	(0.868–0.943)	(0.872–0.947)	(0.853–0.926)
Loneliness	0.773***								
	(0.672–0.888)								
Life satisfaction		1.352***							
		(1.217–1.501)							
Positive affect			1.398***						
			(1.207–1.620)						
Negative affect				0.980					
				(0.844–1.137)					
Optimism					1.361***				
					(1.187–1.560)				
Self-efficacy						1.209*			
						(1.019–1.434)			
Self-esteem							1.290**		
							(1.074–1.550)		
Perceived stress								0.782***	
								(0.695–0.880)	
Self-regulation									0.903
									(0.781–1.044)
Constant	16.10***	3.902***	2.670*	9.483***	3.464**	5.044***	3.993**	17.46***	13.30***
	(7.951–32.59)	(1.965–7.747)	(1.179–6.044)	(4.496–20.00)	(1.642–7.306)	(2.238–11.37)	(1.698–9.386)	(8.591–35.47)	(5.749–30.79)
Observations	7166	7211	7204	7204	7246	7243	7261	7169	7138
Pseudo R^2^	0.064	0.066	0.065	0.063	0.065	0.064	0.064	0.065	0.062

*** *p* < 0.001, ** *p* < 0.01, * *p* < 0.05, + *p* < 0.10; Loneliness [23]; Life satisfaction (SWLS, [25]); Positive and negative affect (PANAS, [26]); Optimism [28]; Self-efficacy [29]; Self-esteem [30]; Self-regulation [33]; Perceived stress [31]

**Table 4:** Correlates of alcohol consumption. Results of multinomial regressions Part 2 (Daily drinkers; Reference category: non-drinker; relative risk ratios were reported, 95% CIs in parentheses)

Independent variables	Daily drinkers	Daily drinkers	Daily drinkers	Daily drinkers	Daily drinkers	Daily drinkers	Daily drinkers	Daily drinkers	Daily drinkers
Female (Ref. Male)	0.229***	0.229***	0.230***	0.231***	0.236***	0.234***	0.231***	0.241***	0.231***
	(0.184–0.284)	(0.185–0.285)	(0.185–0.286)	(0.186–0.287)	(0.190–0.292)	(0.188–0.290)	(0.186–0.287)	(0.194–0.300)	(0.186–0.288)
Age	1.013*	1.011*	1.015**	1.016**	1.015**	1.014**	1.014**	1.013*	1.015**
	(1.003–1.024)	(1.000–1.021)	(1.005–1.026)	(1.005–1.027)	(1.004–1.025)	(1.004–1.025)	(1.004–1.024)	(1.002–1.023)	(1.005–1.026)
Married, living separated from spouse (Ref.: married, living together with spouse)	1.154	1.237	1.131	1.116	1.150	1.123	1.120	1.181	1.096
	(0.520–2.559)	(0.557–2.749)	(0.510–2.508)	(0.504–2.472)	(0.519–2.549)	(0.507–2.489)	(0.506–2.482)	(0.533–2.619)	(0.494–2.432)
Divorced	0.671*	0.749	0.679*	0.671*	0.689*	0.672*	0.679*	0.672*	0.666*
	(0.475–0.947)	(0.529–1.060)	(0.482–0.956)	(0.477–0.945)	(0.489–0.970)	(0.478–0.945)	(0.482–0.955)	(0.477–0.948)	(0.473–0.937)
Widowed	0.775	0.801	0.794	0.800	0.784	0.784	0.789	0.778	0.824
	(0.562–1.069)	(0.582–1.102)	(0.577–1.093)	(0.582–1.101)	(0.570–1.077)	(0.570–1.078)	(0.574–1.084)	(0.564–1.073)	(0.596–1.140)
Single	0.933	1.015	0.932	0.914	0.921	0.906	0.921	0.930	0.865
	(0.627–1.389)	(0.679–1.517)	(0.626–1.386)	(0.615–1.359)	(0.620–1.369)	(0.610–1.345)	(0.620–1.368)	(0.625–1.383)	(0.582–1.288)
Monthly net equivalent income (in €1000)	1.501***	1.478***	1.509***	1.559***	1.516***	1.537***	1.518***	1.472***	1.534***
	(1.348–1.671)	(1.326–1.648)	(1.353–1.683)	(1.398–1.739)	(1.361–1.688)	(1.379–1.712)	(1.362–1.691)	(1.322–1.639)	(1.376–1.710)
East Germany (Ref. West Germany)	0.967	0.968	0.969	0.999	0.984	0.990	0.975	0.960	1.022
	(0.777–1.203)	(0.780–1.202)	(0.781–1.204)	(0.804–1.241)	(0.793–1.221)	(0.798–1.228)	(0.786–1.210)	(0.772–1.194)	(0.822–1.272)
Physical activity: Several times a week (Ref.: daily)	1.614*	1.610*	1.632*	1.621*	1.609*	1.611*	1.609*	1.604*	1.637*
	(1.070–2.436)	(1.070–2.424)	(1.084–2.457)	(1.077–2.439)	(1.069–2.419)	(1.071–2.423)	(1.070–2.419)	(1.063–2.421)	(1.080–2.482)
Once a week	1.926**	1.911**	2.006**	1.918**	1.895**	1.897**	1.901**	1.961**	1.857**
	(1.255–2.956)	(1.248–2.926)	(1.309–3.073)	(1.253–2.935)	(1.239–2.899)	(1.240–2.902)	(1.242–2.907)	(1.277–3.012)	(1.204–2.862)
One to three times a month	2.129**	2.002*	2.052**	1.982*	2.092**	2.079**	2.092**	2.178**	2.120**
	(1.245–3.641)	(1.173–3.417)	(1.202–3.504)	(1.161–3.383)	(1.228–3.562)	(1.221–3.540)	(1.228–3.562)	(1.273–3.725)	(1.235–3.639)
Less frequently	1.467	1.470+	1.533+	1.456	1.481+	1.464	1.474+	1.487+	1.485+
	(0.927–2.320)	(0.931–2.320)	(0.971–2.422)	(0.923–2.297)	(0.938–2.337)	(0.928–2.310)	(0.934–2.326)	(0.940–2.353)	(0.934–2.361)
Never	0.874	0.903	0.959	0.876	0.904	0.880	0.892	0.930	0.871
	(0.592–1.289)	(0.614–1.330)	(0.650–1.415)	(0.596–1.289)	(0.614–1.331)	(0.598–1.294)	(0.606–1.312)	(0.630–1.373)	(0.587–1.293)
Number of physical illnesses	0.918**	0.931*	0.926**	0.900***	0.924**	0.912***	0.920**	0.935*	0.899***
	(0.869–0.970)	(0.881–0.983)	(0.877–0.978)	(0.851–0.951)	(0.874–0.977)	(0.864–0.963)	(0.871–0.972)	(0.884–0.988)	(0.851–0.950)
Loneliness	0.809*								
	(0.671–0.974)								
Life satisfaction		1.320***							
		(1.145–1.522)							
Positive affect			1.504***						
			(1.237–1.829)						
Negative affect				1.132					
				(0.928–1.381)					
Optimism					1.252*				
					(1.042–1.504)				
Self-efficacy						1.118			
						(0.891–1.403)			
Self-esteem							1.297*		
							(1.012–1.664)		
Perceived stress								0.692***	
								(0.590–0.810)	
Self-regulation									0.845+
									(0.696–1.025)
Constant	0.641	0.179***	0.0852***	0.277**	0.194**	0.279*	0.169**	1.038	0.753
	(0.257–1.600)	(0.0716–0.448)	(0.0286–0.254)	(0.105–0.731)	(0.0717–0.525)	(0.0949–0.821)	(0.0530–0.537)	(0.414–2.604)	(0.249–2.273)
Observations	7166	7211	7204	7204	7246	7243	7261	7169	7138
Pseudo R^2^	0.064	0.066	0.065	0.063	0.065	0.064	0.064	0.065	0.062

*** *p* < 0.001, ** *p* < 0.01, * *p* < 0.05, + *p* < 0.10; Loneliness [23]; Life satisfaction (SWLS, [25]); Positive and negative affect (PANAS, [26]); Optimism [28]; Self-efficacy [29]; Self-esteem [30]; Self-regulation [33]; Perceived stress [31]

In all regression models, the number of physical illnesses was negatively associated with a more regular consumption of alcohol (for example, in the model with loneliness as independent variable; occasional drinkers: relative risk ratio = 0.90 [95% CI: 0.87–0.94]; daily drinkers: 0.92 [0.87–0.97]). Furthermore, women drank less frequently alcohol than men (occasional drinkers: 0.72 [0.61–0.84]; daily drinkers: 0.23 [0.18–0.28]). The individual mean net equivalent income per month (in €1000) was positively associated with the occasional drinker status (1.37 [1.24–1.51]) and in particular with the status as daily drinker (1.50 [1.35–1.67]).

With respect to the psychological variables, occasional drinkers and daily drinkers were less affected by loneliness than the never drinking group (occasional drinkers: 0.77 [0.67–0.89]; daily drinkers: 0.81 [0.67–0.97]). Besides, life satisfaction (occasional drinkers: 1.35 [1.22–1.50]; daily drinkers: 1.32 [1.15–1.52]) and positive affect (occasional drinkers: 1.40 [1.21–1.62]; daily drinkers: 1.50 [1.24–1.83]) was higher in both groups. In addition, the level of optimism was higher for occasional (1.36 [1.19–1.56]) and daily drinkers (1.25 [1.04–1.50]) than for never drinkers. The same applies to self-esteem (occasional drinkers: 1.29 [1.07–1.55]; daily drinkers: 1.30 [1.01–1.66]). In contrast, perceived stress was higher for the group of never drinkers as compared to occasional (0.78 [0.70–0.88]) and daily drinkers (0.69 [0.59–0.81]). With respect to self-efficacy, the regression analysis showed a significant difference for occasional drinkers, who were characterized by having a higher self-efficacy as compared to the never drinkers (1.21 [1.02–1.43]), whereas daily drinkers did not differ significantly from this reference group.

Furthermore, differences between occasional and daily drinkers were examined (results not shown, but available upon request). Except for slight differences in perceived stress, which was negatively associated with being a daily drinker, psychological factors did not significantly vary between occasional and daily drinkers. Besides, daily drinking was positively associated with being male, higher income, living in West Germany, and age.

In additional analysis, depression was added to the main model (results not shown). Multinomial regressions showed that compared with non-drinking, occasional and daily drinking were negatively associated with depression. Beyond the association between alcohol consumption and depression, there remained an independent association between alcohol consumption (Ref.: non-drinking) and life satisfaction (occasional drinking; daily drinking), positive affect (occasional drinking; daily drinking), optimism (occasional drinking), and perceived stress (occasional drinking; daily drinking). Furthermore, an association between alcohol consumption and negative affect was observed (daily drinking).

Moreover, regression analysis stratified by age (< 65 years; ≥ 65 years) showed that particularly in individuals aged 40 to 64 years, the association between psychological factors and alcohol consumption was similar in terms of effect sizes and significance, compared with the main model (total sample). This means that loneliness, life satisfaction, positive affect, optimism, self-esteem and perceived stress were significantly associated with alcohol consumption. These results are displayed in the Additional files 1, 2, 3, 4.

Furthermore, we acknowledge the fact that the physical illnesses used in the current study are difficult to compare. Consequently, we performed a sensitivity analysis where all the physical illnesses were entered as dummy-variables in the model. However, results remained virtually the same (not shown, but available upon request).

## Discussion

The aim of the study was to identify factors associated with alcohol intake in older adults, particularly focusing on psychological factors and using a population-based sample of community-dwelling individuals in the second half of life. 11.5% of the individuals never drank alcohol, 76.5% drank alcohol occasionally, and 12.0% drank alcohol every day. After adjusting for various potential confounders, multinomial logistic regressions revealed that in contrast to never drinkers, both occasional and daily drinkers had a decreased loneliness, a higher satisfaction with life, a higher positive affect, a higher optimism, a higher self-efficacy (occasional drinkers only), a higher self-esteem, and less perceived stress. However, as compared with never drinkers, there were no significant differences in negative affect, and self-regulation. Besides, as compared with never drinking, occasional and daily drinking were positively associated with less physical illnesses, male gender, and income.

Except for marked sex differences, daily drinkers were similar regarding the independent variables compared with occasional drinkers. This is in accordance with a random sample of patients from General Practice registers in London (*n* = 241) [38].

Using college student heavy drinkers (*n* = 170; mean age: 19.2 ± 0.8) from a private university in New York, Hustad et al. [7] showed that self-regulation was negatively related with alcohol consumption. Furthermore, Zullig et al. [9] showed that life satisfaction was negatively related with regular alcohol use in sample of South Carolina public high school students in grades 9–12 (*n* = 5032). In contrast, the current study revealed that compared with never drinking, *favorable* levels of the independent variables, like more optimism, a lower illness level or higher self-esteems, were associated with occasional and daily drinking. A possible explanation might be that a large proportion of these non-drinkers are assumed to be ex-drinkers (with high prevalence rates of various chronic conditions), which would be in accordance with previous findings [39, 40]. This might explain why non-drinkers tend to suffer from multiple chronic conditions (see Table 2) as a consequence of sustained drinking that had taken place in the past years, supporting the idea that these individuals are abstinent from alcohol for health reasons (“sick quitter effect”) [40, 41].

In total, the present study is one of the first studies examining factors associated with drinking behavior in the second half of life with a particular focus on psychological factors. Many previous studies focused on adolescents or young adults, whereas only little evidence is available regarding older populations. The latter studies focused in general on a single psychological construct related to the drinking behavior. Our study provides further insights into the relationship of important determinants including various *psychological constructs* using well-established instruments with drinking behavior among *older adults* based on a nationally representative study.

Data from the current study were gathered from a large, representative sample of community-dwelling individuals in the second half of life. The psychological factors examined in our study were operationalized using established and well-accepted measures. In addition, a number of control variables deemed to be important predictors of alcohol intake have been included in the analysis. The present study adds new insights into the relation between factors associated with alcohol intake in older individuals.

The present study also has some limitations that warrant consideration. As indicated by the response rates in the German Ageing Survey, a sample selection bias cannot be ruled out. Thus, it might be difficult to generalize the results to, e.g., individuals with low education or bad subjective health [42]. Moreover, self-rated daily alcohol intake might not fully reflect factual risky alcohol use in the current cross-sectional study, i.e. individuals might not report their drinking accurately. However, it has been demonstrated that self-report methods offer a valid approach to measuring this variable [43, 44]. In addition, the frequency of alcohol consumption does not allow assessing the volume of alcohol intake. Consequently, our data cannot reflect, e.g., binge drinking. Future studies might concentrate on such an outcome measure. Furthermore, it is worth noting that daily alcohol consumptions does not always reflect risky alcohol use. On the contrary, non-daily drinking might sometimes reflect risky drinking. Moreover, our outcome variable (frequency of alcohol consumption) cannot differentiate between non-drinkers and ex-drinkers. This is a main limitation of the current study.

## Conclusions

The current study stresses particularly the relationship between general psychological factors and (risky) alcohol intake in the second half of life. Since interventions are available to address this risk factor [45], this might help to identify individuals with increased alcohol consumption. Upcoming longitudinal studies are required to gain further insights into the determinants of alcohol consumption. Using panel regressions models can help to overcome the shortcomings of cross-sectional observational studies (e.g., self-selection).

## Additional files

Additional file 1: Correlates of alcohol consumption among individuals aged 40 to 64 years. Results of multinomial regressions Part 1 (Occasional drinkers; Reference category: non-drinker; relative risk ratios were reported, 95% CIs in parentheses). (DOCX 18 kb)

Additional file 2: Correlates of alcohol consumption among individuals aged 40 to 64 years. Results of multinomial regressions Part 2 (Daily drinkers; Reference category: non-drinker; relative risk ratios were reported, 95% CIs in parentheses). (DOCX 18 kb)

Additional file 3: Correlates of alcohol consumption among individuals aged 65 years and above. Results of multinomial regressions Part 1 (Occasional drinkers; Reference category: non-drinker; relative risk ratios were reported, 95% CIs in parentheses). (DOCX 18 kb)

Additional file 4: Correlates of alcohol consumption among individuals aged 65 years and above. Results of multinomial regressions Part 2 (Daily drinkers; Reference category: non-drinker; relative risk ratios were reported, 95% CIs in parentheses). (DOCX 17 kb)

## Abbreviations

DEAS: German Ageing Survey
OECD: Organization for Economic Co-Operation and Development
PANAS: Positive and Negative Affect Schedule
SWLS: Satisfaction with Life Scale
