# Supplementary material for: Correlates of alcohol consumption among Germans in the second half of life. Results of a population-based observational study
Source: BMC Geriatr. 2017 Sep 8;17:207. doi: 10.1186/s12877-017-0592-3 (PMC5591529; doi:10.1186/s12877-017-0592-3)
Supplement: Supplementary file 1 — Correlates of alcohol consumption among individuals aged 40 to 64 years. Results of multinomial regressions Part 1 (Occasional drinkers; Reference category: non-drinker; relative risk ratios were reported, 95% CIs in parentheses). (DOCX 18 kb) [file 12877_2017_592_MOESM1_ESM.docx]

Additional file 1. Correlates of alcohol consumption among individuals aged 40 to 64 years. Results of multinomial regressions Part 1 (Occasional drinkers; Reference category: non-drinker; relative risk ratios were reported, 95% CIs in parentheses).

| Independent variables | Occasional drinkers | Occasional drinkers | Occasional drinkers | Occasional drinkers | Occasional drinkers | Occasional drinkers | Occasional drinkers | Occasional drinkers | Occasional drinkers |
| --- | --- | --- | --- | --- | --- | --- | --- | --- | --- |
|  |  |  |  |  |  |  |  |  |  |
| Female (Ref. Male) | 0.903 | 0.921 | 0.931 | 0.955 | 0.943 | 0.953 | 0.948 | 0.959 | 0.958 |
|  | (0.712 - 1.146) | (0.727 - 1.168) | (0.735 - 1.179) | (0.752 - 1.212) | (0.745 - 1.194) | (0.753 - 1.206) | (0.749 - 1.199) | (0.757 - 1.216) | (0.756 - 1.213) |
| Age | 0.980* | 0.979* | 0.982+ | 0.982+ | 0.982+ | 0.982+ | 0.981+ | 0.980* | 0.984+ |
|  | (0.961 - 1.000) | (0.960 - 0.999) | (0.963 - 1.001) | (0.963 - 1.001) | (0.963 - 1.001) | (0.963 - 1.002) | (0.962 - 1.000) | (0.961 - 1.000) | (0.965 - 1.003) |
| Married, living separated from spouse (Ref.: married, living together with spouse) | 1.129 | 1.148 | 1.039 | 1.034 | 1.081 | 1.032 | 1.016 | 1.084 | 0.964 |
|  | (0.498 - 2.564) | (0.507 - 2.600) | (0.460 - 2.346) | (0.459 - 2.327) | (0.478 - 2.447) | (0.458 - 2.325) | (0.451 - 2.289) | (0.481 - 2.444) | (0.429 - 2.169) |
| Divorced | 0.991 | 1.042 | 0.947 | 0.949 | 0.983 | 0.937 | 0.947 | 0.944 | 0.912 |
|  | (0.708 - 1.387) | (0.741 - 1.464) | (0.678 - 1.324) | (0.679 - 1.327) | (0.703 - 1.375) | (0.672 - 1.306) | (0.679 - 1.320) | (0.677 - 1.318) | (0.654 - 1.272) |
| Widowed | 0.763 | 0.779 | 0.752 | 0.755 | 0.768 | 0.751 | 0.766 | 0.786 | 0.735 |
|  | (0.454 - 1.282) | (0.463 - 1.312) | (0.447 - 1.265) | (0.449 - 1.268) | (0.456 - 1.294) | (0.447 - 1.262) | (0.456 - 1.286) | (0.467 - 1.324) | (0.437 - 1.237) |
| Single | 0.729+ | 0.752 | 0.676* | 0.677* | 0.698* | 0.683* | 0.700* | 0.699* | 0.667* |
|  | (0.514 - 1.034) | (0.529 - 1.068) | (0.479 - 0.955) | (0.480 - 0.956) | (0.494 - 0.987) | (0.483 - 0.964) | (0.496 - 0.989) | (0.494 - 0.989) | (0.472 - 0.942) |
| Monthly net equivalent income (in €1,000) | 1.413*** | 1.394*** | 1.445*** | 1.472*** | 1.403*** | 1.451*** | 1.422*** | 1.413*** | 1.463*** |
|  | (1.225 - 1.629) | (1.207 - 1.611) | (1.251 - 1.669) | (1.275 - 1.699) | (1.217 - 1.617) | (1.258 - 1.675) | (1.233 - 1.640) | (1.225 - 1.629) | (1.268 - 1.687) |
| East Germany (Ref. West Germany) | 1.141 | 1.185 | 1.196 | 1.191 | 1.206 | 1.199 | 1.188 | 1.172 | 1.250+ |
|  | (0.889 - 1.466) | (0.924 - 1.519) | (0.934 - 1.533) | (0.928 - 1.528) | (0.941 - 1.546) | (0.936 - 1.535) | (0.927 - 1.522) | (0.914 - 1.503) | (0.973 - 1.604) |
| Physical activity: Several times a week (Ref.: daily) | 1.529+ | 1.564+ | 1.594+ | 1.569+ | 1.578+ | 1.564+ | 1.551+ | 1.538+ | 1.524+ |
|  | (0.945 - 2.476) | (0.966 - 2.533) | (0.986 - 2.577) | (0.970 - 2.536) | (0.975 - 2.552) | (0.968 - 2.526) | (0.960 - 2.506) | (0.951 - 2.487) | (0.942 - 2.464) |
| Once a week | 2.289** | 2.232** | 2.328** | 2.244** | 2.297** | 2.254** | 2.235** | 2.312** | 2.228** |
|  | (1.337 - 3.917) | (1.308 - 3.808) | (1.364 - 3.971) | (1.317 - 3.824) | (1.346 - 3.920) | (1.323 - 3.840) | (1.311 - 3.809) | (1.352 - 3.955) | (1.302 - 3.813) |
| One to three times a month | 1.447 | 1.447 | 1.513 | 1.462 | 1.495 | 1.455 | 1.468 | 1.457 | 1.413 |
|  | (0.803 - 2.605) | (0.804 - 2.602) | (0.841 - 2.720) | (0.814 - 2.625) | (0.831 - 2.689) | (0.810 - 2.614) | (0.817 - 2.639) | (0.810 - 2.620) | (0.785 - 2.541) |
| Less frequently | 1.462 | 1.456 | 1.499 | 1.433 | 1.498 | 1.442 | 1.449 | 1.450 | 1.435 |
|  | (0.868 - 2.462) | (0.865 - 2.449) | (0.891 - 2.524) | (0.853 - 2.409) | (0.890 - 2.522) | (0.858 - 2.423) | (0.862 - 2.436) | (0.862 - 2.439) | (0.851 - 2.419) |
| Never | 0.734 | 0.749 | 0.767 | 0.725 | 0.788 | 0.727 | 0.734 | 0.746 | 0.709 |
|  | (0.460 - 1.172) | (0.469 - 1.195) | (0.480 - 1.227) | (0.455 - 1.156) | (0.493 - 1.260) | (0.456 - 1.159) | (0.461 - 1.171) | (0.467 - 1.191) | (0.444 - 1.132) |
| Number of physical illnesses | 0.887*** | 0.889*** | 0.873*** | 0.865*** | 0.890*** | 0.865*** | 0.881*** | 0.879*** | 0.855*** |
|  | (0.831 - 0.946) | (0.832 - 0.949) | (0.819 - 0.931) | (0.810 - 0.924) | (0.833 - 0.950) | (0.811 - 0.922) | (0.826 - 0.941) | (0.824 - 0.939) | (0.803 - 0.912) |
| Loneliness | 0.652*** |  |  |  |  |  |  |  |  |
|  | (0.532 - 0.800) |  |  |  |  |  |  |  |  |
| Life satisfaction |  | 1.313*** |  |  |  |  |  |  |  |
|  |  | (1.128 - 1.529) |  |  |  |  |  |  |  |
| Positive affect |  |  | 1.270* |  |  |  |  |  |  |
|  |  |  | (1.021 - 1.579) |  |  |  |  |  |  |
| Negative affect |  |  |  | 0.939 |  |  |  |  |  |
|  |  |  |  | (0.760 - 1.161) |  |  |  |  |  |
| Optimism |  |  |  |  | 1.497*** |  |  |  |  |
|  |  |  |  |  | (1.217 - 1.842) |  |  |  |  |
| Self-efficacy |  |  |  |  |  | 1.159 |  |  |  |
|  |  |  |  |  |  | (0.891 - 1.508) |  |  |  |
| Self-esteem |  |  |  |  |  |  | 1.410* |  |  |
|  |  |  |  |  |  |  | (1.084 - 1.832) |  |  |
| Perceived stress |  |  |  |  |  |  |  | 0.767** |  |
|  |  |  |  |  |  |  |  | (0.645 - 0.913) |  |
| Self-regulation |  |  |  |  |  |  |  |  | 0.869 |
|  |  |  |  |  |  |  |  |  | (0.692 - 1.091) |
| Constant | 32.91*** | 5.682** | 5.494* | 14.73*** | 3.875+ | 8.158** | 4.390* | 27.56*** | 21.24*** |
|  | (8.834 - 122.6) | (1.553 - 20.78) | (1.313 - 22.99) | (3.808 - 57.01) | (0.998 - 15.04) | (1.946 - 34.19) | (1.031 - 18.70) | (7.251 - 104.8) | (4.770 - 94.55) |
|  |  |  |  |  |  |  |  |  |  |
| Observations | 3,572 | 3,593 | 3,591 | 3,592 | 3,602 | 3,601 | 3,609 | 3,575 | 3,561 |
| Pseudo R² | 0.072 | 0.071 | 0.069 | 0.069 | 0.072 | 0.069 | 0.070 | 0.071 | 0.068 |

Notes: *** p<0.001, ** p<0.01, * p<0.05, + p<0.10; Loneliness (De Jong Gierveld & Van Tilburg, 2006); Life satisfaction (SWLS, Pavot & Diener, 1993); Positive and negative affect (PANAS, Watson et al., 1988); Optimism (Brandtstädter & Wentura, 1994); Self-efficacy (Schwarzer & Jerusalem, 1999); Self-esteem (Rosenberg, 1965); Self-regulation (Freund & Baltes, 2002); Perceived stress (Cohen et al., 1983), Depression (CES-D≥18, Hautzinger and Bailer, 1993).
