# Supplementary material for: Correlates of alcohol consumption among Germans in the second half of life. Results of a population-based observational study
Source: BMC Geriatr. 2017 Sep 8;17:207. doi: 10.1186/s12877-017-0592-3 (PMC5591529; doi:10.1186/s12877-017-0592-3)
Supplement: Supplementary file 2 — Correlates of alcohol consumption among individuals aged 40 to 64 years. Results of multinomial regressions Part 2 (Daily drinkers; Reference category: non-drinker; relative risk ratios were reported, 95% CIs in parentheses). (DOCX 18 kb) [file 12877_2017_592_MOESM2_ESM.docx]

Additional file 2. Correlates of alcohol consumption among individuals aged 40 to 64 years. Results of multinomial regressions Part 2 (Daily drinkers; Reference category: non-drinker; relative risk ratios were reported, 95% CIs in parentheses).

| Independent variables | Daily drinkers | Daily drinkers | Daily drinkers | Daily drinkers | Daily drinkers | Daily drinkers | Daily drinkers | Daily drinkers | Daily drinkers |
| --- | --- | --- | --- | --- | --- | --- | --- | --- | --- |
|  |  |  |  |  |  |  |  |  |  |
| Female (Ref. Male) | 0.281*** | 0.290*** | 0.291*** | 0.291*** | 0.296*** | 0.298*** | 0.295*** | 0.301*** | 0.302*** |
|  | (0.203 - 0.389) | (0.210 - 0.401) | (0.211 - 0.403) | (0.210 - 0.403) | (0.214 - 0.409) | (0.216 - 0.412) | (0.213 - 0.408) | (0.217 - 0.416) | (0.218 - 0.418) |
| Age | 1.021 | 1.019 | 1.021 | 1.025+ | 1.022 | 1.023+ | 1.021 | 1.020 | 1.022 |
|  | (0.994 - 1.049) | (0.992 - 1.046) | (0.995 - 1.049) | (0.998 - 1.052) | (0.995 - 1.049) | (0.996 - 1.050) | (0.994 - 1.048) | (0.994 - 1.048) | (0.996 - 1.050) |
| Married, living separated from spouse (Ref.: married, living together with spouse) | 1.242 | 1.279 | 1.177 | 1.147 | 1.206 | 1.146 | 1.134 | 1.220 | 1.110 |
|  | (0.428 - 3.605) | (0.441 - 3.710) | (0.407 - 3.402) | (0.398 - 3.308) | (0.417 - 3.489) | (0.397 - 3.307) | (0.393 - 3.272) | (0.422 - 3.526) | (0.384 - 3.205) |
| Divorced | 0.764 | 0.804 | 0.747 | 0.721 | 0.757 | 0.718 | 0.730 | 0.738 | 0.704 |
|  | (0.468 - 1.245) | (0.491 - 1.317) | (0.459 - 1.215) | (0.443 - 1.172) | (0.465 - 1.231) | (0.443 - 1.166) | (0.449 - 1.186) | (0.454 - 1.200) | (0.434 - 1.144) |
| Widowed | 0.817 | 0.853 | 0.829 | 0.813 | 0.847 | 0.821 | 0.832 | 0.850 | 0.811 |
|  | (0.381 - 1.754) | (0.397 - 1.833) | (0.386 - 1.779) | (0.378 - 1.746) | (0.394 - 1.818) | (0.383 - 1.762) | (0.388 - 1.784) | (0.395 - 1.828) | (0.378 - 1.741) |
| Single | 0.725 | 0.754 | 0.688 | 0.678 | 0.703 | 0.685 | 0.703 | 0.702 | 0.672 |
|  | (0.447 - 1.176) | (0.463 - 1.227) | (0.426 - 1.112) | (0.420 - 1.096) | (0.435 - 1.137) | (0.424 - 1.107) | (0.435 - 1.137) | (0.434 - 1.137) | (0.416 - 1.086) |
| Monthly net equivalent income (in €1,000) | 1.536*** | 1.512*** | 1.555*** | 1.600*** | 1.527*** | 1.578*** | 1.546*** | 1.530*** | 1.589*** |
|  | (1.319 - 1.788) | (1.296 - 1.763) | (1.333 - 1.814) | (1.373 - 1.865) | (1.311 - 1.778) | (1.354 - 1.840) | (1.327 - 1.801) | (1.313 - 1.782) | (1.364 - 1.851) |
| East Germany (Ref. West Germany) | 0.940 | 0.974 | 0.980 | 1.009 | 0.982 | 0.986 | 0.975 | 0.965 | 1.011 |
|  | (0.671 - 1.317) | (0.697 - 1.362) | (0.701 - 1.370) | (0.720 - 1.412) | (0.702 - 1.373) | (0.706 - 1.378) | (0.698 - 1.363) | (0.689 - 1.350) | (0.722 - 1.417) |
| Physical activity: Several times a week (Ref.: daily) | 1.825+ | 1.852+ | 1.907+ | 1.861+ | 1.860+ | 1.846+ | 1.835+ | 1.837+ | 1.895+ |
|  | (0.920 - 3.622) | (0.933 - 3.674) | (0.961 - 3.782) | (0.939 - 3.688) | (0.938 - 3.688) | (0.932 - 3.657) | (0.926 - 3.637) | (0.926 - 3.645) | (0.947 - 3.792) |
| Once a week | 2.840** | 2.744** | 2.954** | 2.749** | 2.817** | 2.767** | 2.759** | 2.893** | 2.872** |
|  | (1.357 - 5.945) | (1.314 - 5.729) | (1.413 - 6.178) | (1.318 - 5.735) | (1.349 - 5.881) | (1.326 - 5.774) | (1.322 - 5.758) | (1.382 - 6.054) | (1.359 - 6.067) |
| One to three times a month | 2.107+ | 1.990+ | 2.080+ | 1.940 | 2.106+ | 2.064+ | 2.080+ | 2.138+ | 2.096+ |
|  | (0.943 - 4.706) | (0.890 - 4.447) | (0.928 - 4.663) | (0.867 - 4.340) | (0.944 - 4.699) | (0.926 - 4.599) | (0.933 - 4.640) | (0.958 - 4.773) | (0.932 - 4.715) |
| Less frequently | 1.930+ | 1.908+ | 2.032+ | 1.872+ | 1.959+ | 1.889+ | 1.905+ | 1.900+ | 1.981+ |
|  | (0.926 - 4.023) | (0.916 - 3.975) | (0.974 - 4.240) | (0.900 - 3.895) | (0.941 - 4.081) | (0.908 - 3.930) | (0.915 - 3.965) | (0.911 - 3.963) | (0.941 - 4.168) |
| Never | 1.137 | 1.145 | 1.223 | 1.107 | 1.184 | 1.115 | 1.130 | 1.173 | 1.156 |
|  | (0.573 - 2.253) | (0.578 - 2.270) | (0.616 - 2.430) | (0.559 - 2.189) | (0.596 - 2.350) | (0.563 - 2.205) | (0.571 - 2.237) | (0.591 - 2.325) | (0.577 - 2.313) |
| Number of physical illnesses | 0.934 | 0.938 | 0.932 | 0.895* | 0.935 | 0.910* | 0.929 | 0.933 | 0.905* |
|  | (0.854 - 1.022) | (0.857 - 1.026) | (0.853 - 1.018) | (0.817 - 0.980) | (0.854 - 1.024) | (0.833 - 0.995) | (0.849 - 1.017) | (0.852 - 1.021) | (0.829 - 0.988) |
| Loneliness | 0.652** |  |  |  |  |  |  |  |  |
|  | (0.492 - 0.863) |  |  |  |  |  |  |  |  |
| Life satisfaction |  | 1.310* |  |  |  |  |  |  |  |
|  |  | (1.058 - 1.621) |  |  |  |  |  |  |  |
| Positive affect |  |  | 1.506** |  |  |  |  |  |  |
|  |  |  | (1.117 - 2.030) |  |  |  |  |  |  |
| Negative affect |  |  |  | 1.176 |  |  |  |  |  |
|  |  |  |  | (0.884 - 1.566) |  |  |  |  |  |
| Optimism |  |  |  |  | 1.449* |  |  |  |  |
|  |  |  |  |  | (1.088 - 1.930) |  |  |  |  |
| Self-efficacy |  |  |  |  |  | 1.138 |  |  |  |
|  |  |  |  |  |  | (0.797 - 1.626) |  |  |  |
| Self-esteem |  |  |  |  |  |  | 1.445* |  |  |
|  |  |  |  |  |  |  | (1.002 - 2.083) |  |  |
| Perceived stress |  |  |  |  |  |  |  | 0.701** |  |
|  |  |  |  |  |  |  |  | (0.552 - 0.889) |  |
| Self-regulation |  |  |  |  |  |  |  |  | 0.920 |
|  |  |  |  |  |  |  |  |  | (0.675 - 1.254) |
| Constant | 0.430 | 0.0798** | 0.0405** | 0.112* | 0.0584** | 0.118* | 0.0556** | 0.463 | 0.240 |
|  | (0.0740 - 2.498) | (0.0135 - 0.472) | (0.00568 - 0.288) | (0.0181 - 0.697) | (0.00915 - 0.373) | (0.0167 - 0.828) | (0.00748 - 0.413) | (0.0770 - 2.781) | (0.0316 - 1.817) |
|  |  |  |  |  |  |  |  |  |  |
| Observations | 3,572 | 3,593 | 3,591 | 3,592 | 3,602 | 3,601 | 3,609 | 3,575 | 3,561 |
| Pseudo R² | 0.072 | 0.071 | 0.069 | 0.069 | 0.072 | 0.069 | 0.070 | 0.071 | 0.068 |

Notes: *** p<0.001, ** p<0.01, * p<0.05, + p<0.10; Loneliness (De Jong Gierveld & Van Tilburg, 2006); Life satisfaction (SWLS, Pavot & Diener, 1993); Positive and negative affect (PANAS, Watson et al., 1988); Optimism (Brandtstädter & Wentura, 1994); Self-efficacy (Schwarzer & Jerusalem, 1999); Self-esteem (Rosenberg, 1965); Self-regulation (Freund & Baltes, 2002); Perceived stress (Cohen et al., 1983), Depression (CES-D≥18, Hautzinger and Bailer, 1993).
