# Supplementary material for: Correlates of alcohol consumption among Germans in the second half of life. Results of a population-based observational study
Source: BMC Geriatr. 2017 Sep 8;17:207. doi: 10.1186/s12877-017-0592-3 (PMC5591529; doi:10.1186/s12877-017-0592-3)
Supplement: Supplementary file 3 — Correlates of alcohol consumption among individuals aged 65 years and above. Results of multinomial regressions Part 1 (Occasional drinkers; Reference category: non-drinker; relative risk ratios were reported, 95% CIs in parentheses). (DOCX 18 kb) [file 12877_2017_592_MOESM3_ESM.docx]

Additional file 3. Correlates of alcohol consumption among individuals aged 65 years and above. Results of multinomial regressions Part 1 (Occasional drinkers; Reference category: non-drinker; relative risk ratios were reported, 95% CIs in parentheses).

| Independent variables | Occasional drinkers | Occasional drinkers | Occasional drinkers | Occasional drinkers | Occasional drinkers | Occasional drinkers | Occasional drinkers | Occasional drinkers | Occasional drinkers |
| --- | --- | --- | --- | --- | --- | --- | --- | --- | --- |
|  |  |  |  |  |  |  |  |  |  |
| Female (Ref. Male) | 0.574*** | 0.574*** | 0.570*** | 0.583*** | 0.593*** | 0.582*** | 0.580*** | 0.597*** | 0.560*** |
|  | (0.461 - 0.714) | (0.462 - 0.713) | (0.459 - 0.709) | (0.469 - 0.724) | (0.478 - 0.735) | (0.469 - 0.722) | (0.468 - 0.719) | (0.480 - 0.742) | (0.450 - 0.698) |
| Age | 0.993 | 0.986 | 0.993 | 0.989 | 0.991 | 0.991 | 0.991 | 0.995 | 0.993 |
|  | (0.975 - 1.011) | (0.968 - 1.004) | (0.975 - 1.011) | (0.971 - 1.007) | (0.973 - 1.009) | (0.974 - 1.009) | (0.973 - 1.009) | (0.977 - 1.014) | (0.975 - 1.011) |
| Married, living separated from spouse (Ref.: married, living together with spouse) | 0.897 | 0.971 | 0.867 | 0.895 | 0.907 | 0.893 | 0.899 | 0.904 | 0.806 |
|  | (0.333 - 2.414) | (0.359 - 2.624) | (0.323 - 2.333) | (0.333 - 2.405) | (0.337 - 2.442) | (0.332 - 2.406) | (0.334 - 2.419) | (0.336 - 2.431) | (0.297 - 2.187) |
| Divorced | 0.657* | 0.740+ | 0.643* | 0.653* | 0.674* | 0.669* | 0.669* | 0.661* | 0.647* |
|  | (0.469 - 0.920) | (0.526 - 1.043) | (0.459 - 0.902) | (0.467 - 0.914) | (0.481 - 0.943) | (0.479 - 0.936) | (0.478 - 0.936) | (0.472 - 0.925) | (0.461 - 0.907) |
| Widowed | 0.809 | 0.821 | 0.805 | 0.815 | 0.807 | 0.809 | 0.818 | 0.802+ | 0.860 |
|  | (0.624 - 1.051) | (0.633 - 1.064) | (0.621 - 1.044) | (0.628 - 1.057) | (0.623 - 1.045) | (0.625 - 1.047) | (0.632 - 1.059) | (0.618 - 1.042) | (0.660 - 1.120) |
| Single | 2.087* | 2.672** | 2.462* | 2.338* | 2.178* | 2.180* | 2.153* | 2.093* | 2.037* |
|  | (1.031 - 4.224) | (1.266 - 5.640) | (1.170 - 5.179) | (1.114 - 4.907) | (1.077 - 4.404) | (1.078 - 4.409) | (1.065 - 4.351) | (1.034 - 4.236) | (1.006 - 4.124) |
| Monthly net equivalent income (in €1,000) | 1.308*** | 1.300*** | 1.309*** | 1.360*** | 1.339*** | 1.340*** | 1.334*** | 1.277*** | 1.326*** |
|  | (1.133 - 1.510) | (1.123 - 1.504) | (1.132 - 1.515) | (1.174 - 1.576) | (1.158 - 1.548) | (1.160 - 1.549) | (1.155 - 1.542) | (1.107 - 1.473) | (1.146 - 1.535) |
| East Germany (Ref. West Germany) | 1.257* | 1.212+ | 1.221+ | 1.248* | 1.241+ | 1.250* | 1.237+ | 1.243+ | 1.248* |
|  | (1.007 - 1.567) | (0.974 - 1.507) | (0.981 - 1.519) | (1.003 - 1.554) | (1.000 - 1.542) | (1.006 - 1.552) | (0.996 - 1.537) | (0.998 - 1.550) | (1.001 - 1.557) |
| Physical activity: Several times a week (Ref.: daily) | 0.915** | 0.936* | 0.932** | 0.916** | 0.932* | 0.923** | 0.922** | 0.930** | 0.913*** |
|  | (0.868 - 0.965) | (0.887 - 0.987) | (0.883 - 0.983) | (0.867 - 0.967) | (0.883 - 0.984) | (0.875 - 0.973) | (0.874 - 0.972) | (0.881 - 0.981) | (0.866 - 0.963) |
| Once a week | 1.501+ | 1.447+ | 1.463+ | 1.459+ | 1.457+ | 1.458+ | 1.471+ | 1.496+ | 1.448+ |
|  | (1.000 - 2.254) | (0.967 - 2.165) | (0.978 - 2.189) | (0.975 - 2.182) | (0.975 - 2.176) | (0.976 - 2.177) | (0.985 - 2.196) | (0.996 - 2.247) | (0.959 - 2.189) |
| One to three times a month | 1.040 | 1.066 | 1.116 | 1.070 | 1.052 | 1.062 | 1.055 | 1.054 | 1.001 |
|  | (0.694 - 1.558) | (0.712 - 1.595) | (0.746 - 1.670) | (0.716 - 1.600) | (0.705 - 1.569) | (0.712 - 1.585) | (0.707 - 1.574) | (0.703 - 1.580) | (0.666 - 1.506) |
| Less frequently | 2.552** | 2.358* | 2.400* | 2.396* | 2.435** | 2.400* | 2.432** | 2.584** | 2.498** |
|  | (1.279 - 5.092) | (1.207 - 4.605) | (1.229 - 4.686) | (1.228 - 4.676) | (1.248 - 4.751) | (1.230 - 4.680) | (1.247 - 4.742) | (1.295 - 5.155) | (1.247 - 5.001) |
| Never | 0.905 | 0.930 | 0.951 | 0.929 | 0.940 | 0.937 | 0.939 | 0.916 | 0.908 |
|  | (0.561 - 1.457) | (0.578 - 1.497) | (0.591 - 1.531) | (0.578 - 1.494) | (0.585 - 1.511) | (0.583 - 1.505) | (0.585 - 1.510) | (0.568 - 1.475) | (0.559 - 1.474) |
| Number of physical illnesses | 0.628** | 0.653* | 0.704+ | 0.644* | 0.659* | 0.643* | 0.646* | 0.653* | 0.615** |
|  | (0.441 - 0.894) | (0.459 - 0.928) | (0.494 - 1.004) | (0.453 - 0.914) | (0.464 - 0.936) | (0.453 - 0.913) | (0.455 - 0.916) | (0.458 - 0.931) | (0.429 - 0.881) |
| Loneliness | 0.911 |  |  |  |  |  |  |  |  |
|  | (0.751 - 1.104) |  |  |  |  |  |  |  |  |
| Life satisfaction |  | 1.361*** |  |  |  |  |  |  |  |
|  |  | (1.175 - 1.577) |  |  |  |  |  |  |  |
| Positive affect |  |  | 1.547*** |  |  |  |  |  |  |
|  |  |  | (1.262 - 1.897) |  |  |  |  |  |  |
| Negative affect |  |  |  | 1.014 |  |  |  |  |  |
|  |  |  |  | (0.821 - 1.253) |  |  |  |  |  |
| Optimism |  |  |  |  | 1.244* |  |  |  |  |
|  |  |  |  |  | (1.035 - 1.494) |  |  |  |  |
| Self-efficacy |  |  |  |  |  | 1.229+ |  |  |  |
|  |  |  |  |  |  | (0.981 - 1.541) |  |  |  |
| Self-esteem |  |  |  |  |  |  | 1.150 |  |  |
|  |  |  |  |  |  |  | (0.888 - 1.490) |  |  |
| Perceived stress |  |  |  |  |  |  |  | 0.796** |  |
|  |  |  |  |  |  |  |  | (0.676 - 0.937) |  |
| Self-regulation |  |  |  |  |  |  |  |  | 0.943 |
|  |  |  |  |  |  |  |  |  | (0.780 - 1.140) |
| Constant | 14.03*** | 5.556* | 2.386 | 14.00*** | 6.404* | 6.396* | 7.901* | 16.49*** | 15.24*** |
|  | (3.228 - 60.98) | (1.283 - 24.07) | (0.466 - 12.23) | (3.108 - 63.06) | (1.387 - 29.56) | (1.279 - 31.99) | (1.500 - 41.62) | (3.897 - 69.76) | (3.040 - 76.36) |
|  |  |  |  |  |  |  |  |  |  |
| Observations | 3,594 | 3,618 | 3,613 | 3,612 | 3,644 | 3,642 | 3,652 | 3,594 | 3,577 |
| Pseudo R² | 0.059 | 0.062 | 0.062 | 0.059 | 0.060 | 0.059 | 0.059 | 0.060 | 0.059 |

Notes: *** p<0.001, ** p<0.01, * p<0.05, + p<0.10; Loneliness (De Jong Gierveld & Van Tilburg, 2006); Life satisfaction (SWLS, Pavot & Diener, 1993); Positive and negative affect (PANAS, Watson et al., 1988); Optimism (Brandtstädter & Wentura, 1994); Self-efficacy (Schwarzer & Jerusalem, 1999); Self-esteem (Rosenberg, 1965); Self-regulation (Freund & Baltes, 2002); Perceived stress (Cohen et al., 1983), Depression (CES-D≥18, Hautzinger and Bailer, 1993).
