# Supplementary material for: Correlates of alcohol consumption among Germans in the second half of life. Results of a population-based observational study
Source: BMC Geriatr. 2017 Sep 8;17:207. doi: 10.1186/s12877-017-0592-3 (PMC5591529; doi:10.1186/s12877-017-0592-3)
Supplement: Supplementary file 4 — Correlates of alcohol consumption among individuals aged 65 years and above. Results of multinomial regressions Part 2 (Daily drinkers; Reference category: non-drinker; relative risk ratios were reported, 95% CIs in parentheses). (DOCX 17 kb) [file 12877_2017_592_MOESM4_ESM.docx]

Additional file 4. Correlates of alcohol consumption among individuals aged 65 years and above. Results of multinomial regressions Part 2 (Daily drinkers; Reference category: non-drinker; relative risk ratios were reported, 95% CIs in parentheses).

| Independent variables | Daily drinkers | Daily drinkers | Daily drinkers | Daily drinkers | Daily drinkers | Daily drinkers | Daily drinkers | Daily drinkers | Daily drinkers |
| --- | --- | --- | --- | --- | --- | --- | --- | --- | --- |
|  |  |  |  |  |  |  |  |  |  |
| Female (Ref. Male) | 0.185*** | 0.181*** | 0.181*** | 0.182*** | 0.186*** | 0.184*** | 0.182*** | 0.193*** | 0.178*** |
|  | (0.137 - 0.248) | (0.135 - 0.243) | (0.134 - 0.243) | (0.135 - 0.245) | (0.138 - 0.249) | (0.137 - 0.246) | (0.136 - 0.244) | (0.144 - 0.260) | (0.132 - 0.239) |
| Age | 1.001 | 0.994 | 1.001 | 0.998 | 0.998 | 0.999 | 0.999 | 1.003 | 1.000 |
|  | (0.977 - 1.025) | (0.971 - 1.018) | (0.977 - 1.025) | (0.974 - 1.022) | (0.975 - 1.022) | (0.975 - 1.022) | (0.975 - 1.022) | (0.980 - 1.028) | (0.976 - 1.024) |
| Married, living separated from spouse (Ref.: married, living together with spouse) | 1.048 | 1.131 | 1.023 | 1.046 | 1.056 | 1.055 | 1.057 | 1.081 | 1.024 |
|  | (0.312 - 3.517) | (0.336 - 3.806) | (0.305 - 3.427) | (0.312 - 3.510) | (0.315 - 3.538) | (0.314 - 3.540) | (0.315 - 3.545) | (0.322 - 3.623) | (0.304 - 3.453) |
| Divorced | 0.588* | 0.687 | 0.609* | 0.617+ | 0.626+ | 0.626+ | 0.626+ | 0.603* | 0.633+ |
|  | (0.360 - 0.958) | (0.421 - 1.122) | (0.375 - 0.990) | (0.381 - 1.002) | (0.386 - 1.015) | (0.386 - 1.014) | (0.386 - 1.015) | (0.370 - 0.982) | (0.390 - 1.029) |
| Widowed | 0.846 | 0.889 | 0.877 | 0.894 | 0.869 | 0.874 | 0.873 | 0.838 | 0.938 |
|  | (0.588 - 1.216) | (0.620 - 1.274) | (0.612 - 1.258) | (0.623 - 1.283) | (0.607 - 1.244) | (0.610 - 1.251) | (0.610 - 1.250) | (0.583 - 1.205) | (0.650 - 1.354) |
| Single | 2.565* | 3.200** | 2.979* | 2.901* | 2.601* | 2.609* | 2.593* | 2.565* | 2.395* |
|  | (1.124 - 5.851) | (1.352 - 7.576) | (1.263 - 7.028) | (1.232 - 6.829) | (1.141 - 5.931) | (1.144 - 5.949) | (1.137 - 5.911) | (1.124 - 5.853) | (1.043 - 5.500) |
| Monthly net equivalent income (in €1,000) | 1.439*** | 1.433*** | 1.442*** | 1.500*** | 1.474*** | 1.476*** | 1.466*** | 1.394*** | 1.456*** |
|  | (1.235 - 1.677) | (1.226 - 1.674) | (1.235 - 1.685) | (1.283 - 1.754) | (1.263 - 1.719) | (1.265 - 1.721) | (1.257 - 1.710) | (1.197 - 1.623) | (1.246 - 1.700) |
| East Germany (Ref. West Germany) | 0.985 | 0.959 | 0.959 | 0.989 | 0.980 | 0.987 | 0.975 | 0.953 | 1.017 |
|  | (0.737 - 1.317) | (0.721 - 1.277) | (0.720 - 1.277) | (0.742 - 1.319) | (0.737 - 1.302) | (0.743 - 1.312) | (0.733 - 1.296) | (0.714 - 1.272) | (0.762 - 1.359) |
| Physical activity: Several times a week (Ref.: daily) | 1.500 | 1.477 | 1.487 | 1.481 | 1.481 | 1.483 | 1.483 | 1.480 | 1.516 |
|  | (0.885 - 2.543) | (0.876 - 2.490) | (0.882 - 2.507) | (0.879 - 2.495) | (0.880 - 2.493) | (0.881 - 2.496) | (0.881 - 2.496) | (0.873 - 2.510) | (0.889 - 2.586) |
| Once a week | 1.529 | 1.546 | 1.605+ | 1.548 | 1.510 | 1.522 | 1.518 | 1.550 | 1.444 |
|  | (0.901 - 2.597) | (0.913 - 2.617) | (0.948 - 2.719) | (0.914 - 2.620) | (0.894 - 2.551) | (0.900 - 2.572) | (0.898 - 2.566) | (0.912 - 2.635) | (0.845 - 2.467) |
| One to three times a month | 2.880* | 2.582* | 2.618* | 2.593* | 2.716* | 2.694* | 2.704* | 2.908* | 2.898* |
|  | (1.278 - 6.489) | (1.166 - 5.719) | (1.182 - 5.798) | (1.171 - 5.741) | (1.230 - 5.996) | (1.221 - 5.948) | (1.225 - 5.968) | (1.291 - 6.553) | (1.280 - 6.559) |
| Less frequently | 1.157 | 1.171 | 1.196 | 1.167 | 1.173 | 1.172 | 1.173 | 1.183 | 1.162 |
|  | (0.628 - 2.128) | (0.638 - 2.151) | (0.651 - 2.196) | (0.636 - 2.143) | (0.640 - 2.152) | (0.639 - 2.149) | (0.639 - 2.151) | (0.643 - 2.179) | (0.626 - 2.156) |
| Never | 0.777 | 0.816 | 0.866 | 0.793 | 0.806 | 0.795 | 0.799 | 0.829 | 0.768 |
|  | (0.482 - 1.253) | (0.508 - 1.310) | (0.537 - 1.395) | (0.494 - 1.274) | (0.502 - 1.293) | (0.495 - 1.276) | (0.498 - 1.283) | (0.513 - 1.338) | (0.473 - 1.249) |
| Number of physical illnesses | 0.917* | 0.931* | 0.927* | 0.907** | 0.923* | 0.917* | 0.919* | 0.939+ | 0.902** |
|  | (0.855 - 0.983) | (0.868 - 0.998) | (0.865 - 0.994) | (0.845 - 0.974) | (0.860 - 0.990) | (0.856 - 0.983) | (0.858 - 0.986) | (0.875 - 1.008) | (0.842 - 0.968) |
| Loneliness | 0.966 |  |  |  |  |  |  |  |  |
|  | (0.750 - 1.244) |  |  |  |  |  |  |  |  |
| Life satisfaction |  | 1.330** |  |  |  |  |  |  |  |
|  |  | (1.095 - 1.616) |  |  |  |  |  |  |  |
| Positive affect |  |  | 1.520** |  |  |  |  |  |  |
|  |  |  | (1.167 - 1.978) |  |  |  |  |  |  |
| Negative affect |  |  |  | 1.102 |  |  |  |  |  |
|  |  |  |  | (0.836 - 1.452) |  |  |  |  |  |
| Optimism |  |  |  |  | 1.109 |  |  |  |  |
|  |  |  |  |  | (0.871 - 1.413) |  |  |  |  |
| Self-efficacy |  |  |  |  |  | 1.077 |  |  |  |
|  |  |  |  |  |  | (0.800 - 1.449) |  |  |  |
| Self-esteem |  |  |  |  |  |  | 1.139 |  |  |
|  |  |  |  |  |  |  | (0.809 - 1.603) |  |  |
| Perceived stress |  |  |  |  |  |  |  | 0.694*** |  |
|  |  |  |  |  |  |  |  | (0.560 - 0.861) |  |
| Self-regulation |  |  |  |  |  |  |  |  | 0.816 |
|  |  |  |  |  |  |  |  |  | (0.636 - 1.048) |
| Constant | 1.535 | 0.731 | 0.310 | 1.392 | 1.171 | 1.249 | 1.029 | 2.637 | 3.568 |
|  | (0.230 - 10.26) | (0.108 - 4.938) | (0.0369 - 2.602) | (0.200 - 9.689) | (0.160 - 8.554) | (0.155 - 10.09) | (0.117 - 9.030) | (0.409 - 17.02) | (0.445 - 28.62) |
|  |  |  |  |  |  |  |  |  |  |
| Observations | 3,594 | 3,618 | 3,613 | 3,612 | 3,644 | 3,642 | 3,652 | 3,594 | 3,577 |
| Pseudo R² | 0.059 | 0.062 | 0.062 | 0.059 | 0.060 | 0.059 | 0.0590 | 0.060 | 0.059 |

Notes: *** p<0.001, ** p<0.01, * p<0.05, + p<0.10; Loneliness (De Jong Gierveld & Van Tilburg, 2006); Life satisfaction (SWLS, Pavot & Diener, 1993); Positive and negative affect (PANAS, Watson et al., 1988); Optimism (Brandtstädter & Wentura, 1994); Self-efficacy (Schwarzer & Jerusalem, 1999); Self-esteem (Rosenberg, 1965); Self-regulation (Freund & Baltes, 2002); Perceived stress (Cohen et al., 1983), Depression (CES-D≥18, Hautzinger and Bailer, 1993).
